# Crystal structure of *cis*-*anti*-*cis*-di­cyclo­hexane-18-crown-6 aceto­nitrile disolvate

**DOI:** 10.1107/S2056989015011056

**Published:** 2015-06-13

**Authors:** Alexander Nazarenko

**Affiliations:** a Chemistry Department, SUNY College at Buffalo, 1300 Elmwood Ave, Buffalo, New York 14222, USA

**Keywords:** crystal structure, di­cyclohexane-18-crown-6, crown ether, aceto­nitrile, hydrogen bonding

## Abstract

The title compound (systematic name: *cis*-*anti*-*cis*-2,5,8,15,18,21-hexa­oxatri­cyclo­[20.4.0.0^9,14^]hexa­cosane aceto­nitrile disolvate), C_20_H_36_O_6_·2CH_3_CN, crystallizes from an aceto­nitrile solution of di­cyclo­hexane-18-crown-6 on evaporation. The mol­ecule is arranged around a center of symmetry with half the crown ether mol­ecule and one mol­ecule of aceto­nitrile symmetry independent. All O—C—C—O torsion angles are *gauche* while all C—O—C—C angles are *trans*. The sequence of torsion angles is [(*tg^+^t*)(*tg^−^t*)]_3_; the geometry of oxygen atoms is close to pseudo-*D*_3*d*_ with three atoms below and three atoms above the mean plane, with an average deviation of ±0.16 (1) Å from the mean plane. This geometry is identical to that observed in metal ion complexes of di­cyclo­hexane-18-crown-6 but differs significantly from the conformation of a free unsolvated mol­ecule. Each aceto­nitrile mol­ecule connects to a crown ether mol­ecule *via* two of its methyl group H atoms (C—H⋯O). Weaker inter­actions exist between the third H atom of the aceto­nitrile methyl group and an O atom of a neighbouring crown ether mol­ecule (C—H⋯O); and between the N atom of the aceto­nitrile mol­ecule and a H atom of another neighbouring crown ether mol­ecule. All these inter­molecular inter­actions create a three-dimensional network stabilizing the disolvate.

## Related literature   

The crystal structure of the *cis*-*anti*-*cis* isomer of di­cyclo­hexane-18-crown-6 was reported by Dalley *et al.* (1975 ▸) (no atomic coordinates given), and later re-investigated by Naza­renko (2002 ▸). For the ortho­rhom­bic polymorph, see: Kravtsov *et al.* (2002 ▸). Synthesis and crystal structures of solvates of di­cyclo­hexane-18-crown-6 with di­nitriles have been investigated; see: structures with malono­nitrile by Damewood *et al.* (1988 ▸) and with succino­nitrile by Dalley & Naza­renko (1999 ▸). The importance of the different behavior of isomers of di­cyclo­hexane-18-crown-6 was first stressed by Pedersen (1967 ▸) and later studied in complexation, extraction, and transport reactions.
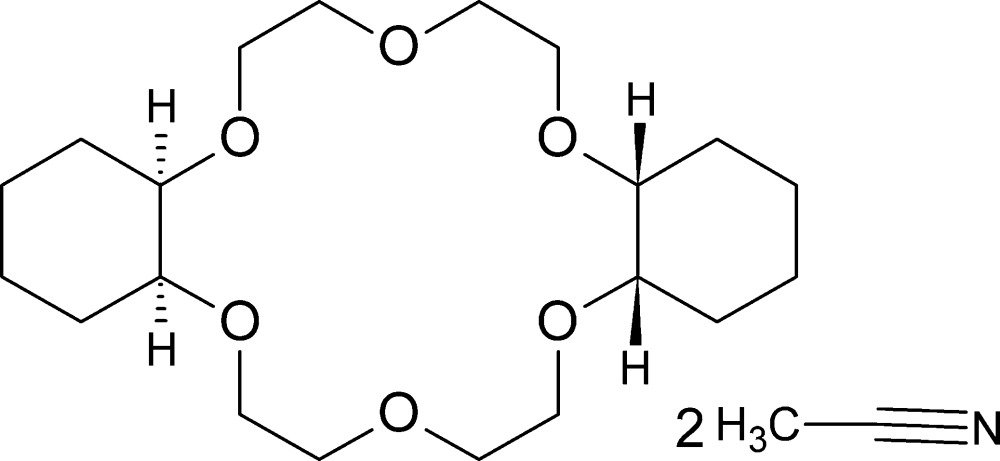


## Experimental   

### Crystal data  

C_20_H_36_O_6_·2C_2_H_3_N
*M* *_r_* = 454.59
Triclinic, 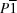

*a* = 6.9428 (4) Å
*b* = 9.5286 (5) Å
*c* = 9.8927 (6) Å
α = 80.415 (2)°
β = 81.697 (2)°
γ = 80.927 (2)°
*V* = 632.53 (6) Å^3^
*Z* = 1
Mo *K*α radiation
μ = 0.09 mm^−1^
*T* = 173 K
0.49 × 0.34 × 0.28 mm

### Data collection  

Bruker PHOTON-100 CMOS diffractometer
Absorption correction: multi-scan (*SADABS*; Bruker, 2014 ▸) *T*_min_ = 0.956, *T*_max_ = 1.000
20203 measured reflections
3045 independent reflections
2476 reflections with *I* > 2σ(*I*)
*R*_int_ = 0.031

### Refinement  

*R*[*F* ^2^ > 2σ(*F* ^2^)] = 0.041
*wR*(*F* ^2^) = 0.104
*S* = 1.07
3045 reflections
229 parameters
All H-atom parameters refined
Δρ_max_ = 0.29 e Å^−3^
Δρ_min_ = −0.19 e Å^−3^

### 

Data collection: *APEX2* (Bruker, 2013 ▸); cell refinement: *SAINT* (Bruker, 2013 ▸); data reduction: *SAINT*; program(s) used to solve structure: *SHELXT* (Sheldrick, 2015*a* ▸); program(s) used to refine structure: *SHELXL2014* (Sheldrick, 2015*b* ▸); molecular graphics: *OLEX2* (Dolomanov *et al.*, 2009 ▸); software used to prepare material for publication: *OLEX2*.

## Supplementary Material

Crystal structure: contains datablock(s) I. DOI: 10.1107/S2056989015011056/zl2627sup1.cif

Structure factors: contains datablock(s) I. DOI: 10.1107/S2056989015011056/zl2627Isup2.hkl

Supporting information file. DOI: 10.1107/S2056989015011056/zl2627Isup3.cdx

**Fig. 1.**
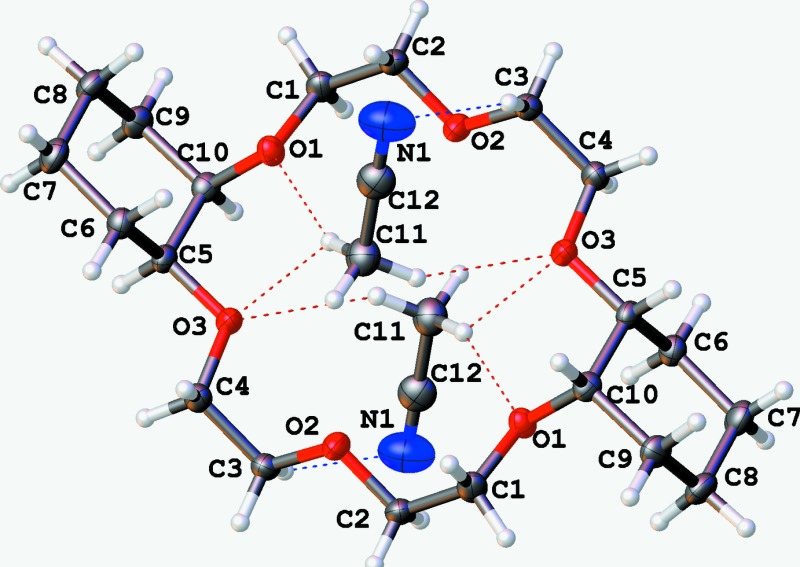
Structure of the title compound with atom labeling. The second half of the crown ether molecule and the second acetonitrile molecule molecule are created by an inversion center located at the center of the crown ether molecule (symmetry operator: 1 - x, 1 - y, 1 - z).

Supporting information file. DOI: 10.1107/S2056989015011056/zl2627Isup4.cml

x z . DOI: 10.1107/S2056989015011056/zl2627fig1.tif
Structure of the title compound with atom labeling. The second half of the crown ether mol­ecule and the second aceto­nitrile mol­ecule mol­ecule are created by an inversion center located at the center of the crown ether mol­ecule (symmetry operator: 1 − *x*, 1 − y, 1 − *z*).

**Fig. 2.**
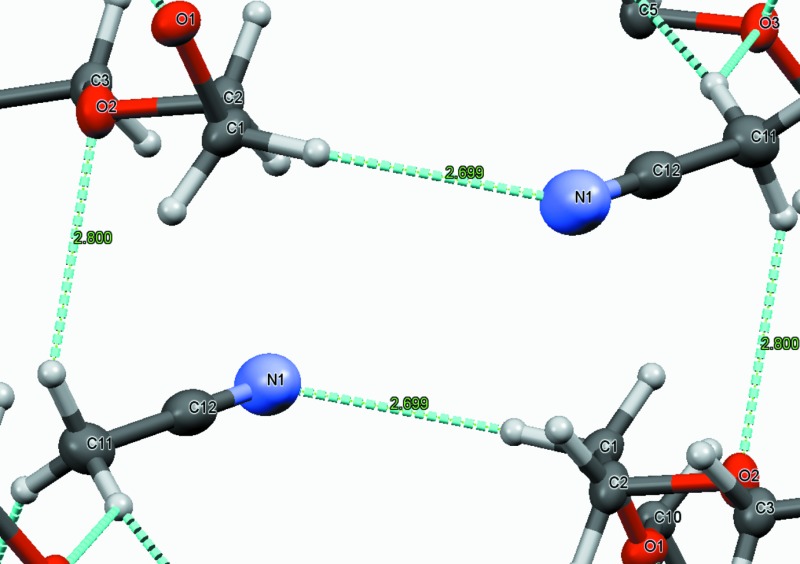
Intermolecular short contacts of acetonitrile molecules with neighboring crown ether molecules.

. DOI: 10.1107/S2056989015011056/zl2627fig2.tif
Inter­molecular short contacts of aceto­nitrile mol­ecules with neighboring crown ether mol­ecules.

CCDC reference: 1405283

Additional supporting information:  crystallographic information; 3D view; checkCIF report

## Figures and Tables

**Table 1:** Hydrogen-bond geometry (, )

*D*H*A*	*D*H	H*A*	*D* *A*	*D*H*A*
C11H11*A*O3^i^	0.936(19)	2.51(2)	3.3528(17)	150.8(16)
C11H11*C*O1^i^	0.97(2)	2.56(2)	3.4809(16)	159.1(15)

Symmetry code: (i) 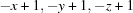.

## supplementary crystallographic information

### Crystal data

**Table:**

C_20_H_36_O_6_·2C_2_H_3_N	*Z* = 1
*M**_r_* = 454.59	*F*(000) = 248
Triclinic, *P*1	*D*_x_ = 1.193 Mg m^−^^3^
*a* = 6.9428 (4) Å	Mo *K*α radiation, λ = 0.71073 Å
*b* = 9.5286 (5) Å	Cell parameters from 9918 reflections
*c* = 9.8927 (6) Å	θ = 3.0–33.2°
α = 80.415 (2)°	µ = 0.09 mm^−^^1^
β = 81.697 (2)°	*T* = 173 K
γ = 80.927 (2)°	Block, colourless
*V* = 632.53 (6) Å^3^	0.49 × 0.34 × 0.28 mm

### Data collection

**Table:**

Bruker PHOTON-100 CMOS diffractometer	2476 reflections with *I* > 2σ(*I*)
Detector resolution: 10 pixels mm^-1^	*R*_int_ = 0.031
φ and ω scans	θ_max_ = 28.0°, θ_min_ = 3.0°
Absorption correction: multi-scan (*SADABS*; Bruker, 2014)	*h* = −9→9
*T*_min_ = 0.956, *T*_max_ = 1.000	*k* = −12→12
20203 measured reflections	*l* = −13→13
3045 independent reflections	

### Refinement

**Table:**

Refinement on *F*^2^	Primary atom site location: structure-invariant direct methods
Least-squares matrix: full	Hydrogen site location: difference Fourier map
*R*[*F*^2^ > 2σ(*F*^2^)] = 0.041	All H-atom parameters refined
*wR*(*F*^2^) = 0.104	*w* = 1/[σ^2^(*F*_o_^2^) + (0.0454*P*)^2^ + 0.1761*P*] where *P* = (*F*_o_^2^ + 2*F*_c_^2^)/3
*S* = 1.07	(Δ/σ)_max_ < 0.001
3045 reflections	Δρ_max_ = 0.29 e Å^−^^3^
229 parameters	Δρ_min_ = −0.19 e Å^−^^3^
0 restraints	

### Special details

**Table:**

Experimental. SADABS-2014/5 (Bruker, 2014) was used for absorption correction. wR2(int) was 0.0615 before and 0.0513 after correction. The Ratio of minimum to maximum transmission is 0.9562. The λ/2 correction factor is 0.00150.
Geometry. All e.s.d.'s (except the e.s.d. in the dihedral angle between two l.s. planes) are estimated using the full covariance matrix. The cell e.s.d.'s are taken into account individually in the estimation of e.s.d.'s in distances, angles and torsion angles; correlations between e.s.d.'s in cell parameters are only used when they are defined by crystal symmetry. An approximate (isotropic) treatment of cell e.s.d.'s is used for estimating e.s.d.'s involving l.s. planes.

### Fractional atomic coordinates and isotropic or equivalent isotropic displacement parameters (Å^2^)

**Table:**

	*x*	*y*	*z*	*U*_iso_*/*U*_eq_	
O3	0.32768 (12)	0.49496 (8)	0.76820 (8)	0.0246 (2)	
O1	0.47462 (11)	0.27282 (8)	0.32574 (8)	0.02307 (19)	
O2	0.24108 (12)	0.30700 (8)	0.59392 (8)	0.0258 (2)	
C10	0.50905 (17)	0.66821 (12)	0.81733 (11)	0.0222 (2)	
C5	0.31527 (17)	0.60704 (12)	0.84980 (11)	0.0217 (2)	
C2	0.28223 (19)	0.18001 (13)	0.53071 (12)	0.0265 (3)	
C1	0.29308 (18)	0.22030 (14)	0.37680 (13)	0.0275 (3)	
C4	0.16935 (18)	0.41295 (13)	0.80064 (13)	0.0259 (3)	
C3	0.22641 (18)	0.27499 (12)	0.73991 (12)	0.0244 (3)	
C6	0.14216 (17)	0.72519 (13)	0.82817 (13)	0.0245 (2)	
C9	0.50976 (19)	0.78031 (13)	0.91167 (13)	0.0278 (3)	
C8	0.3383 (2)	0.90062 (13)	0.89292 (14)	0.0312 (3)	
C7	0.1431 (2)	0.84186 (14)	0.91736 (14)	0.0319 (3)	
C12	0.25068 (19)	0.76690 (15)	0.34942 (15)	0.0356 (3)	
N1	0.2335 (2)	0.87640 (16)	0.28169 (17)	0.0589 (4)	
C11	0.2721 (2)	0.62716 (15)	0.43442 (15)	0.0346 (3)	
H5	0.3067 (19)	0.5644 (13)	0.9475 (14)	0.021 (3)*	
H6A	0.1510 (18)	0.7665 (14)	0.7324 (14)	0.022 (3)*	
H8A	0.357 (2)	0.9525 (15)	0.7989 (16)	0.032 (4)*	
H1A	0.183 (2)	0.2961 (15)	0.3529 (14)	0.031 (4)*	
H2A	0.408 (2)	0.1246 (15)	0.5572 (15)	0.032 (4)*	
H10	0.618 (2)	0.5904 (14)	0.8319 (13)	0.024 (3)*	
H3A	0.356 (2)	0.2254 (14)	0.7689 (13)	0.023 (3)*	
H9A	0.499 (2)	0.7318 (15)	1.0061 (16)	0.032 (4)*	
H2B	0.179 (2)	0.1217 (15)	0.5635 (14)	0.029 (3)*	
H4A	0.140 (2)	0.3869 (15)	0.9035 (16)	0.031 (4)*	
H3B	0.124 (2)	0.2113 (14)	0.7749 (14)	0.026 (3)*	
H6B	0.015 (2)	0.6841 (15)	0.8527 (14)	0.028 (3)*	
H9B	0.636 (2)	0.8182 (15)	0.8941 (15)	0.032 (4)*	
H7A	0.121 (2)	0.8002 (16)	1.0119 (17)	0.035 (4)*	
H1B	0.281 (2)	0.1362 (17)	0.3363 (15)	0.036 (4)*	
H8B	0.337 (2)	0.9694 (16)	0.9556 (16)	0.038 (4)*	
H4B	0.053 (2)	0.4659 (16)	0.7635 (15)	0.033 (4)*	
H7B	0.034 (2)	0.9160 (17)	0.9006 (16)	0.040 (4)*	
H11A	0.360 (3)	0.5628 (19)	0.3858 (19)	0.055 (5)*	
H11B	0.147 (3)	0.5936 (19)	0.4572 (19)	0.057 (5)*	
H11C	0.326 (3)	0.635 (2)	0.517 (2)	0.069 (6)*	

### Atomic displacement parameters (Å^2^)

**Table:**

	*U*^11^	*U*^22^	*U*^33^	*U*^12^	*U*^13^	*U*^23^
O3	0.0272 (4)	0.0223 (4)	0.0253 (4)	−0.0080 (3)	0.0043 (3)	−0.0085 (3)
O1	0.0231 (4)	0.0282 (4)	0.0189 (4)	−0.0070 (3)	−0.0018 (3)	−0.0040 (3)
O2	0.0344 (5)	0.0210 (4)	0.0222 (4)	−0.0053 (3)	0.0006 (3)	−0.0055 (3)
C10	0.0249 (6)	0.0223 (5)	0.0196 (5)	−0.0013 (4)	−0.0055 (4)	−0.0028 (4)
C5	0.0283 (6)	0.0208 (5)	0.0163 (5)	−0.0032 (4)	−0.0013 (4)	−0.0047 (4)
C2	0.0308 (6)	0.0222 (6)	0.0276 (6)	−0.0095 (5)	0.0034 (5)	−0.0072 (5)
C1	0.0263 (6)	0.0324 (6)	0.0273 (6)	−0.0117 (5)	−0.0003 (5)	−0.0099 (5)
C4	0.0262 (6)	0.0266 (6)	0.0257 (6)	−0.0081 (5)	0.0030 (5)	−0.0072 (5)
C3	0.0286 (6)	0.0229 (6)	0.0222 (6)	−0.0086 (5)	0.0009 (5)	−0.0034 (4)
C6	0.0241 (6)	0.0259 (6)	0.0237 (6)	−0.0022 (4)	−0.0010 (4)	−0.0065 (5)
C9	0.0335 (7)	0.0308 (6)	0.0221 (6)	−0.0079 (5)	−0.0054 (5)	−0.0078 (5)
C8	0.0408 (7)	0.0249 (6)	0.0303 (7)	−0.0046 (5)	−0.0029 (5)	−0.0119 (5)
C7	0.0329 (7)	0.0292 (6)	0.0335 (7)	0.0011 (5)	0.0009 (5)	−0.0137 (5)
C12	0.0277 (7)	0.0402 (8)	0.0411 (8)	−0.0068 (5)	−0.0073 (5)	−0.0080 (6)
N1	0.0539 (9)	0.0474 (8)	0.0755 (11)	−0.0121 (7)	−0.0197 (7)	0.0062 (7)
C11	0.0317 (7)	0.0371 (7)	0.0340 (7)	−0.0009 (6)	−0.0032 (6)	−0.0063 (6)

### Geometric parameters (Å, º)

**Table:**

O3—C5	1.4274 (13)	C4—H4B	0.969 (15)
O3—C4	1.4169 (14)	C3—H3A	1.005 (13)
O1—C10^i^	1.4285 (13)	C3—H3B	0.993 (14)
O1—C1	1.4230 (14)	C6—C7	1.5313 (16)
O2—C2	1.4241 (13)	C6—H6A	0.961 (13)
O2—C3	1.4177 (14)	C6—H6B	1.006 (14)
C10—O1^i^	1.4285 (13)	C9—C8	1.5256 (18)
C10—C5	1.5210 (16)	C9—H9A	0.969 (15)
C10—C9	1.5319 (16)	C9—H9B	0.983 (15)
C10—H10	0.982 (13)	C8—C7	1.5196 (19)
C5—C6	1.5248 (16)	C8—H8A	0.980 (15)
C5—H5	0.981 (13)	C8—H8B	0.972 (16)
C2—C1	1.5009 (17)	C7—H7A	0.953 (16)
C2—H2A	0.994 (15)	C7—H7B	0.964 (16)
C2—H2B	0.964 (15)	C12—N1	1.1414 (19)
C1—H1A	0.992 (15)	C12—C11	1.450 (2)
C1—H1B	0.974 (16)	C11—H11A	0.934 (19)
C4—C3	1.5086 (16)	C11—H11B	0.959 (19)
C4—H4A	1.005 (15)	C11—H11C	0.97 (2)
			
C4—O3—C5	114.40 (8)	O2—C3—H3B	110.1 (8)
C1—O1—C10^i^	114.05 (8)	C4—C3—H3A	109.8 (7)
C3—O2—C2	111.74 (9)	C4—C3—H3B	108.8 (8)
O1^i^—C10—C5	106.49 (9)	H3A—C3—H3B	109.1 (10)
O1^i^—C10—C9	112.88 (9)	C5—C6—C7	110.51 (10)
O1^i^—C10—H10	108.5 (8)	C5—C6—H6A	109.1 (8)
C5—C10—C9	109.33 (9)	C5—C6—H6B	110.4 (8)
C5—C10—H10	109.5 (8)	C7—C6—H6A	109.7 (8)
C9—C10—H10	110.0 (8)	C7—C6—H6B	109.2 (8)
O3—C5—C10	107.36 (9)	H6A—C6—H6B	107.8 (11)
O3—C5—C6	114.17 (9)	C10—C9—H9A	107.7 (8)
O3—C5—H5	108.6 (7)	C10—C9—H9B	110.2 (8)
C10—C5—C6	110.98 (9)	C8—C9—C10	110.82 (10)
C10—C5—H5	106.1 (8)	C8—C9—H9A	110.1 (9)
C6—C5—H5	109.4 (7)	C8—C9—H9B	111.4 (8)
O2—C2—C1	109.38 (10)	H9A—C9—H9B	106.6 (12)
O2—C2—H2A	109.3 (8)	C9—C8—H8A	108.6 (8)
O2—C2—H2B	109.0 (8)	C9—C8—H8B	110.4 (9)
C1—C2—H2A	110.7 (8)	C7—C8—C9	111.38 (11)
C1—C2—H2B	109.7 (8)	C7—C8—H8A	109.9 (8)
H2A—C2—H2B	108.7 (11)	C7—C8—H8B	109.5 (9)
O1—C1—C2	109.04 (10)	H8A—C8—H8B	107.0 (12)
O1—C1—H1A	109.4 (8)	C6—C7—H7A	108.0 (9)
O1—C1—H1B	111.4 (9)	C6—C7—H7B	108.9 (9)
C2—C1—H1A	110.0 (8)	C8—C7—C6	111.85 (10)
C2—C1—H1B	108.9 (9)	C8—C7—H7A	108.5 (9)
H1A—C1—H1B	108.1 (12)	C8—C7—H7B	112.3 (9)
O3—C4—C3	109.11 (9)	H7A—C7—H7B	106.9 (13)
O3—C4—H4A	110.3 (8)	N1—C12—C11	179.47 (16)
O3—C4—H4B	110.7 (9)	C12—C11—H11A	109.2 (11)
C3—C4—H4A	107.7 (8)	C12—C11—H11B	109.6 (11)
C3—C4—H4B	109.4 (9)	C12—C11—H11C	108.9 (12)
H4A—C4—H4B	109.6 (12)	H11A—C11—H11B	109.9 (15)
O2—C3—C4	109.08 (9)	H11A—C11—H11C	108.5 (17)
O2—C3—H3A	110.0 (7)	H11B—C11—H11C	110.8 (16)
			
O3—C5—C6—C7	−178.84 (9)	C5—C10—C9—C8	−58.39 (13)
O3—C4—C3—O2	−68.55 (12)	C5—C6—C7—C8	54.14 (14)
O1^i^—C10—C5—O3	62.59 (10)	C2—O2—C3—C4	−176.21 (10)
O1^i^—C10—C5—C6	−62.81 (11)	C4—O3—C5—C10	171.48 (9)
O1^i^—C10—C9—C8	59.93 (13)	C4—O3—C5—C6	−65.05 (12)
O2—C2—C1—O1	75.52 (12)	C3—O2—C2—C1	179.16 (10)
C10^i^—O1—C1—C2	−172.39 (9)	C9—C10—C5—O3	−175.17 (9)
C10—C5—C6—C7	−57.34 (13)	C9—C10—C5—C6	59.43 (12)
C10—C9—C8—C7	55.90 (14)	C9—C8—C7—C6	−53.72 (15)
C5—O3—C4—C3	−163.53 (9)		

Symmetry code: (i) −*x*+1, −*y*+1, −*z*+1.

### Hydrogen-bond geometry (Å, º)

**Table:**

*D*—H···*A*	*D*—H	H···*A*	*D*···*A*	*D*—H···*A*
C11—H11*A*···O3^i^	0.936 (19)	2.51 (2)	3.3528 (17)	150.8 (16)
C11—H11*C*···O1^i^	0.97 (2)	2.56 (2)	3.4809 (16)	159.1 (15)

Symmetry code: (i) −*x*+1, −*y*+1, −*z*+1.
