# Supplementary figures and images for: Crystal structure of cis-anti-cis-di­cyclo­hexane-18-crown-6 aceto­nitrile disolvate
Source: Acta Crystallogr E Crystallogr Commun. 2015 Jun 13;71(Pt 7):o472–3. doi: 10.1107/S2056989015011056 (PMC4518961; doi:10.1107/S2056989015011056)

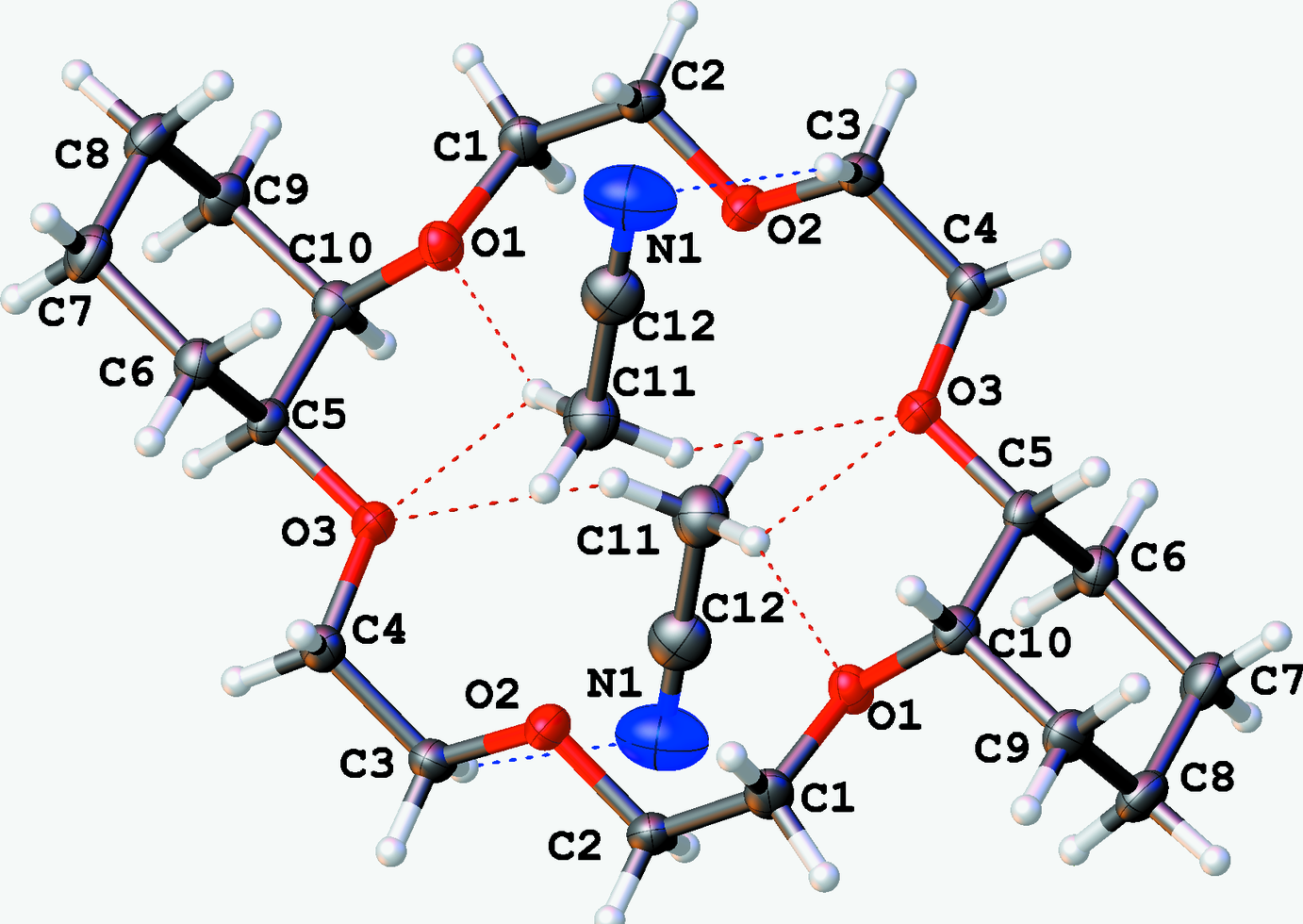

Supplement: Supplementary file 5 [file e-71-0o472-fig1.tif]

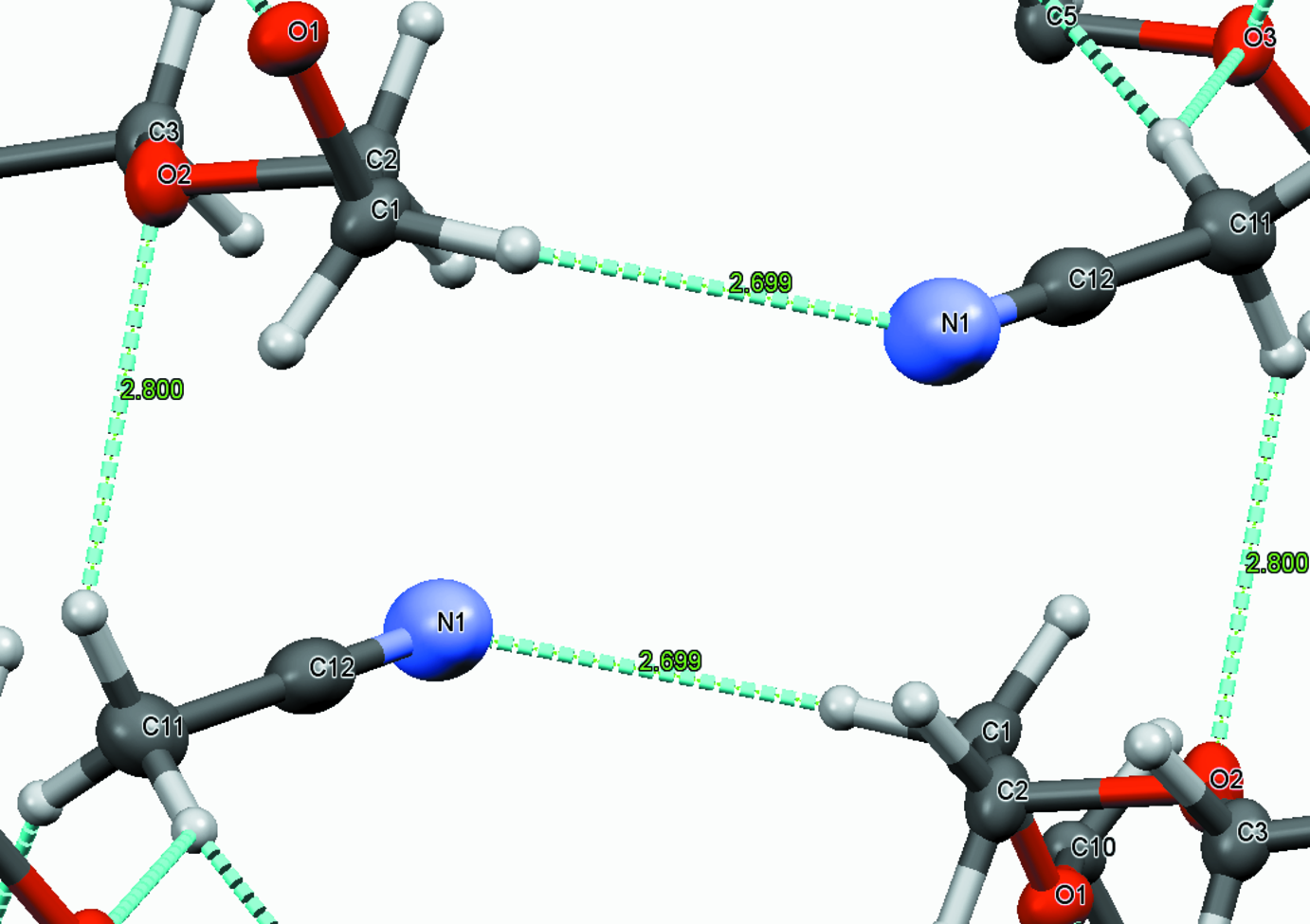

Supplement: Supplementary file 6 [file e-71-0o472-fig2.tif]
